# Fast compressive Raman micro-spectroscopy to image and classify microplastics from natural marine environment

**DOI:** 10.1016/j.eti.2024.103622

**Published:** 2024-05

**Authors:** Clément Grand, Camille Scotté, Énora Prado, Maria El Rakwe, Olivier Fauvarque, Hervé Rigneault

**Affiliations:** a Aix Marseille Univ, CNRS, Centrale Med, Institut Fresnel, Marseille, France; b INRAE, UMR ITAP, 361 Rue Jean François Breton, Montpellier 34090, France; c Ifremer, RDT Research and Technological Development, Plouzané 29280, France

**Keywords:** Microplastics, Microplastics detection, Raman imaging, Compressive Raman, Raman spectroscopy

## Abstract

The fast and reliable detection of micron-sized plastic particles from the natural marine environment is an important topic that is mostly addressed using spontaneous Raman spectroscopy. Due to the long (>tens of ms) integration time required to record a viable Raman signal, measurements are limited to a single point per microplastic particle or require very long acquisition times (up to tens of hours). In this work, we develop, validate, and demonstrate a compressive Raman technology using binary spectral filters and single-pixel detection that can image and classify six types of marine microplastic particles over an area of 1 mm^2^ with a pixel dwell time down to 1.75 ms/pixel and a spatial resolution of 1 µm. This is x10–100 faster than reported in previous studies.

## Highlights

• Compressive Raman is a technology that utilizes binary filters to conduct Raman spectroscopy with a single photodetector.
• The technology can image, detect, and classify six different types of microplastics found in the natural marine environment.
• Compressive Raman offers a tenfold speed improvement compared to conventional spontaneous Raman spectroscopy.

## Graphical Abstract

**Figure:**
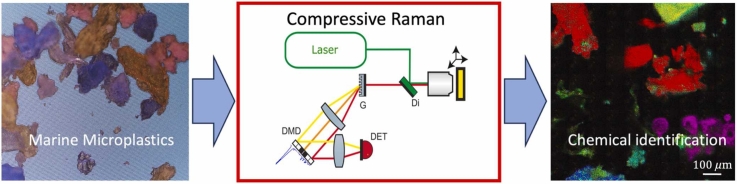

## 1 Introduction

Left in the environment, plastics degrade according to kinetics that depend on abiotic (ultraviolet rays, oxygen, water, etc.) and biotic (microorganisms) factors. The degradation of plastics results in their fragmentation into small particles. When these are smaller than 5 mm, they are called microplastics (MPs). Nano-plastics (which size is between 1 nm and 1 *µ*m) are also released throughout the aging of plastics by erosion of their surface, altered on its first micrometers, in particular under the effect of oxidation. From visible pollution, plastic pollution becomes invisible when it comes to micro and nano-plastics. As a result, animal species very easily ingest large quantities of plastic of various sizes, which their digestive systems cannot absorb, resulting to internal burns, digestive obstructions, and even death (Kühn et al., 2015). Additionally, toxins from ingested plastic have been shown to harm reproduction and weaken the immune system of animals (Kühn et al., 2015). The human species is of course not spared and there is a lack of data on the impact of plastic pollution on diets (Senathirajah et al., 2021) with concerns concerning the amount of plastic ingested by humans every day.

Solutions to plastic pollution must be found in areas such as health, food and the environment. In addition, sources of plastic are varied and modified by additives, ranging from textiles and tire dust to bottles and packaging. Therefore, in order to know *how* and *where* to intervene effectively, whatever the area concerned, a realistic assessment based on representative data of the quality and quantity of the MPs responsible for this pollution must be established.

Conventional Raman spectroscopy is one of the main methods used for the identification of very small MPs (< 20 µm) (Araujo et al., 2018), along with FTIR (Fourier transform infrared spectroscopy) (Song et al., 2015, Hidalgo-Ruz et al., 2012) and GC/MS (Gas chromatography-mass spectrometry) (Fries et al., 2013, Chen et al., 2020). Among the optical techniques, conventional Raman spectroscopy seems to be the most suitable for a complete study of the MP range (from 5 mm to 1 µm) since it allow µm resolution (Chen et al., 2020). In addition to being a non-destructive and label-free method, Raman spectrometry requires minimal sample preparation with highly specific spectra and negligible interference from water, allowing direct analysis of aqueous samples (Vašková, 2022, Ivleva et al., 2017). Identifications of plastic families have already been carried out by Raman spectroscopy, whether in sediments (Van Cauwenberghe et al., 2013), in the oceans (Lusher et al., 2014, Enders et al., 2015) or in marine organisms (Van Cauwenberghe and Janssen, 2014, Cole et al., 2013, Goldstein and Goodwin, 2013). MPs identification by stimulated Raman scattering (SRS) has been also recently demonstrated with acquisition speeds >100 times faster (a few µs pixel dwell time) than conventional Raman mapping (typically ms pixel dwell time) on samples of the same type (Zada et al., 2018, Rigneault and Berto, sept. 2018). Furthermore, SRS is not affected by autofluorescence, which is often present in environmental samples (Laptenok et al., 2020) but comes with a drastic increase in technological complexity (ultra-fast lasers, complex detection scheme, …) (Sarri et al., 2019, Heuke et al., 2021) and financial cost. MPs fieldwork can benefit from a less expensive and easier-to-use tool. In this paper a fast spontaneous Raman imaging technique known as compressive Raman technology (CRT), is used to perform fast detection (<500 *µ*s pixel dwell time), fast identification and quantification of MPs. Six types of marine microplastic particles collected on a silicon filter were imaged and classified over an area of 1 mm^2^ and with a 1 *µ*m spatial resolution for a total acquisition time of less than two hours.

**Fig. 1:**
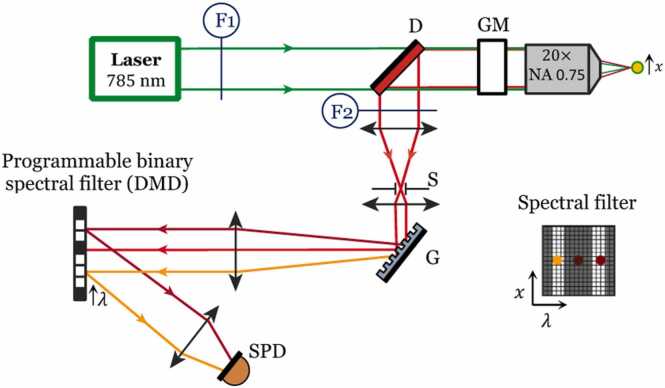
Simplified sketch of the developed NIR CRT setup (see also Fig. S1); D: dichroic mirror; GM: Galvanometric mirrors; S: confocal slit; G: amplitude grating (1200 lines/mm); DMD: digital micro mirror device; SPD: single pixel detector; F1: laser line filter (LL01-785–12.5, Semrock), F2: notch filter.

Ideally, Raman analysis should provide spatially resolved maps for the MPs studied. However, Raman spectroscopy at some specific sample locations associated with bright-filed microscopy is often preferred over Raman imaging. This is because the acquisition of the complete Raman spectrum for each MPs spatial pixel, coupled with the weak Raman scattering cross section and detector array noise, requires lengthy acquisition times and generates large data sets (Nolan and Sebba, 2011, Panczer et al., 2022). This is especially true when studying plastics taken from the natural environment, which are often combined with additives (giving a color to certain plastics depending on what they are made from). Indeed, this type of sample is known in the literature to emit a lot of background noise (fluorescence background) sometimes requiring much more acquisition time (Araujo et al., 2018, Ribeiro Claro et al., 2016) and signal post processing. Typically, MPs studies using spontaneous Raman imaging requires a >0.5 s pixel dwell time that lead to long acquisition time (> 25 hours up few days) to image surface of mm² with a spatial resolution of ∼1 µm (Araujo et al., 2018, Sobhani et al., 2019, Käppler et al., 2015). Notably, acquiring the complete Raman spectra at each sample pixel is a very inefficient way to map the spatial distribution of known chemical compounds. A more efficient way takes advantage of the knowledge of the molecular compounds that are present in a sample (Scotté et al., 2018, Cebeci et al., 2013). This precisely what does the recently developed compressive Raman technology (CRT) (Wilcox et al., 2012, Wilcox et al., 2013, Grand et al., 2022, Scotté et al., 2023, Rehrauer et al., 2018, Réfrégier et al., 2018). In CRT the measurement is directly designed to estimate the quantities of interest (e.g., molecular concentrations), rather than deducing them from complete Raman spectra acquired at each pixel. Owing to the photon noise detection limit of single pixel detector and to the ability to estimate the concentrations of chemical species with a low number of photons, CRT has shown to perform Raman images ×10 to ×100 times faster than conventional EMCCD and CCD based Raman systems (Scotté et al., 2018). Fig. 1 shows a schematic view of a compressive Raman microscope (Sturm et al., 2019) where a fast programmable binary spectral filter, is inserted into a spectrometer to send selected Raman photons onto a single pixel detector (Scotté et al., 2018, Wilcox et al., 2012). The design of these binary spectral filters and the algorithms on which they are based will be described in greater detail below.

This paper follows the classification method reported in (Réfrégier et al., 2019) to map and classify 7 different types of MPs. It is demonstrated here that these chemical species can be successfully detected and identified at each micrometer-sized location on the surface of a laboratory coverslip or a micrometer filter at a pixel integration time as low as 250 *µ*s per species. This allows a 1 mm² area to be mapped in 30 minutes (4 min per species) with a spatial resolution of 1 *µ*m.

## 2 Material and methods

### 2.1 Compressive Raman technology (CRT)

The complete CRT setup is shown in supporting information figure S1 and described earlier in (Grand et al., 2022, Grand, 2023), a simplified sketch is provided in Fig. 1. It uses a 785 nm CW laser (IPS – D-Type Module −100 mW – 100 MHz bandwidth) together with a 3 nm bandwidth (48 cm^−1^) laser line filter at 785 nm (Semrock 785 nm MaxLine). A two-axis galvanometric scanner (Cambridge Technology) was used to steer the beam together with a commercial microscope stand (Nikon Eclipse) equipped with a ×20, NA = 0.75 objective lens (Nikon CFI Apo Lambda). This objective focuses the 785 nm excitation light and collects the back emitted/reflected Raman photons. The latter are de-scanned and separated from the excitation beam by a dichroic mirror and sent to a custom-built CRT spectrometer. At the spectrometer entrance a 785 nm notch filter (HSPF785.0 – Kaiser) rejects all the remaining laser and Rayleigh light. The CRT spectrometer is composed of a high transmission (∼85%) grating (1200 lines/mm) that disperses the Raman photons towards a dispersive micromirror device (DMD Vialux V-650 L NIR) with optimized NIR reflection. The binary encoded spectral filters displayed on the DMD send the Raman photons towards a single photon avalanche photodiode (SPAD-ID Quantique-ID120) with a 75% quantum efficiency at 800 nm. The galvo mirrors and image acquisition are controlled by a custom LabView based software. This software controls also the DMD display via the Vialux interface. The spectral resolution of the CRT spectrometer is estimated to be δλ ≈ 12 cm^−1^ and the spatial resolution 1 *µ*m (x,y) and 10 *µ*m (z axis) (measured with calibrated samples, data not shown).

### 2.2 CRT algorithm

#### 2.2.1 Filter design

Central to CRT are the design of the binary filters that are displayed sequentially to classify chemical species, at each pixel (see supp info paragraph 3). The reported algorithms (Wilcox et al., 2012, Réfrégier et al., 2018) proceed in a similar way to design filters that maximize the precision of the chemical species proportions. This is done by the minimization of the trace of covariance (Wilcox et al., 2012), or the Cramer-Rao lower bound (Réfrégier et al., 2018), matrices. The two approaches being equivalent when the number of binary filters equals the number of chemical species (Réfrégier et al., 2018). The filter design is based on the Cramer-Rao lower bound given by (Réfrégier et al., 2018, Scotté, 2022).

#### 2.2.2 Classification

The CRT classification algorithm is based on the work from Réfrégier et al (2019).; it assumes that one pixel of an image (size 1 µm) represents only one species. From the obtained filtered measurements, this algorithm compares for each pixel the probability of each known species to be present. Among these different probabilities, the classifier chooses the species with the highest probability of identification for a given pixel and associates the respective color (see supp info paragraph 3).

In the case of classification, it is assumed that there is a single species per pixel (which is the case for MP particle that are equal or bigger than the 1 µm CRT microscope pixel size). A probability of error must be taken into consideration here. It will be limited by the bound of Bhattacharyya (BB). This bound is calculated from the reference spectra and the matched filters. BB is therefore greater than the probability of error of the classification method. Its limits lead to a simple expression of a minimum number of photons necessary to increase the probability of error. With this, the color corresponding to the pixel class can be displayed directly, but this is far from a "physical" image since the intensities do not appear. Since it is assumed that there is only one species, the weighting of the color of each pixel by the total number of photons received on the pixel is applied. Indeed since in this case, all the photons are supposed to come from the unique species of the pixel. A detailed analysis of the BB bound in CRT classification can be found in (Réfrégier et al., 2019).

#### 2.2.3 MP from the marine environment

**Fig. 2:**
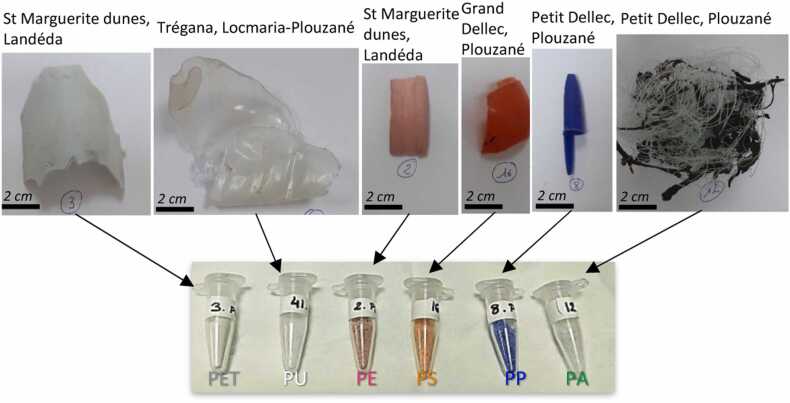
Macroplastic wastes found in different Britany area (France) before and after cryo-grinding. The specific localities are precised on the top. Eppendorf samples from left to right: MPs sample of polyethylene terephthalate (PET, uncolored), polyurethane (PU, white), polyethylene (PE, pink), polystyrene (PS, orange), polypropylene (PP, blue) and Nylon (PA, light green).

**Fig. 3:**
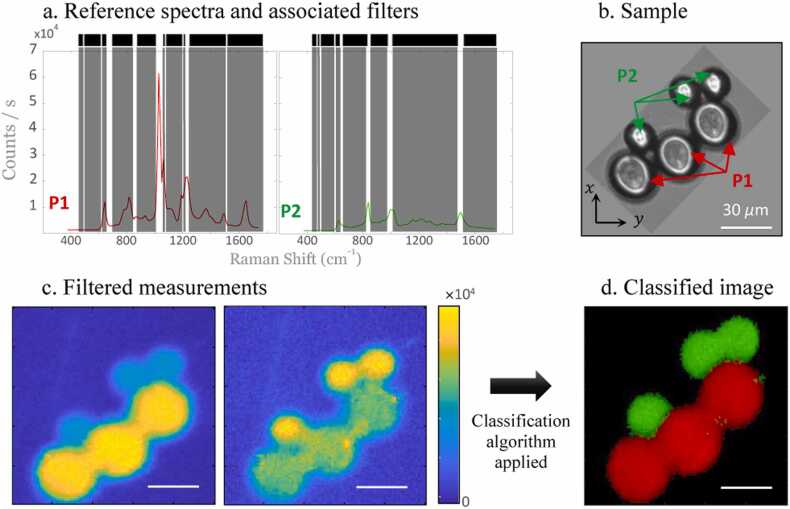
(a) Raman spectra of P1 (polystyrene beads, 30 *µ*m diameter) and P2 (methyl methacrylate beads, 20 *µ*m diameter) with the superimposed designed binary filters (Raman photons falling in the white stripes are kept while the photons falling in the grey stripes are rejected), (b) sample seen from a microscope camera. (c) projection map of the two different spectral filters from the image seen in (b) where the exposure time is 250 *µ*s/pix/filter. (d) Classification map after applying the algorithm, described in (Réfrégier et al., 2019), on the filtered measurements shown in (c). For each image, the scale bar is 30 *µ*m and the FOV is 100 *µ*m × 100 *µ*m (100 pixels × 100 pixels).

The samples were collected from natural marine environments in Britany area (France) from natural marine environments (sea, beaches, etc.) (Fig. 2). They were macroplastics with sizes ranging from a few mm to a few cm. These raw samples have undergone minimal initial treatments before their analysis with Raman spectroscopy. These treatments consisted in manually removing foreign macroelements present in the harvest samples. If necessary, the plastics were washed with filtered water to remove any sand and salt residue to be finally crushed (cryo-grinding) to produce plastic microparticles of different types (and colors) and different sizes. The polymers were identified by Raman spectroscopy before and after fragmentation, no polymers fingerprints variations were observed (result not show). Each type of plastic polymer has a different color caused by its own (unknown) additives that were originally added to it during its manufacture (Fig. 3). The figure S2 compares Raman spectra of these different plastic polymers from a conventional Raman spectrometer with those from a compressed Raman spectrometer.

## 3 Results

### 3.1 MPs test beads images with CRT

To illustrate the operation and relevance of CRT for MPs imaging, a simple model is chosen involving only two types of synthetic MPs beads deposited on a CaF_2_ coverslip that are labelled P1 (polystyrene beads, 30 *µ*m diameter) and P2 (methyl methacrylate beads, 20 *µ*m diameter) (Sigma Aldrich) (Fig. 3(b)). Spontaneous Raman spectra of P1 and P2 - measured with the Raman spectrometer of Fig. 1- are shown in Fig. 3(a). These known spectra are used to design binary filters that minimize the variance of the estimated P1 and P2 proportions following (Scotté et al., 2018, Réfrégier et al., 2018). The designed binary filters are presented on the respective Raman spectra for P1 and P2 (Raman photons falling in the white bands are detected while photons falling in the grey bands are rejected). Note that a spectral filter constructed from this model adapts not only to a spectral species but also to the combination of spectra involved in the experiment. In general, a filter cannot be associated to a specific specie, this is the combination of the signals that are coming from each filter that enables the identification of the species, at each spatial point. Once the sample is scanned for each spectral filter displayed on the DMD, the filtered measurements are obtained (Fig. 3(c)) where the scale is proportional to the photon count per pixel. Note that this process is also done for the background (CaF_2_) spectral filter which is also considered as a species.

The CRT classification algorithm (based on the work from Réfrégier et al. (2019).) assumes that one pixel of an image (size 1 *µ*m) represents only one species. From the obtained filtered measurements, this algorithm compares for each pixel the probability of each known species to be present. Among these different probabilities, the classifier chooses the species with the highest probability of identification for a given pixel and associates the respective color: P1 (red), P2 (green) or background (black). This classified image provides a colored image of the two distributions of P1 and P2 (Fig. 3(d)). Note that no other image processing (smoothing…) was performed to improve the image rendering.

### 3.2 CRT imaging of mixed MPs samples from the marine environment

**Fig. 4:**
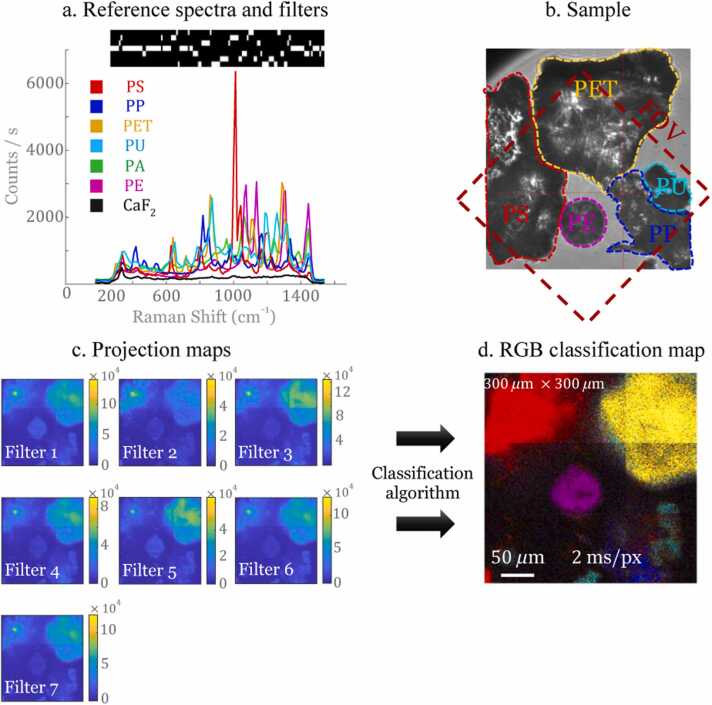
(a) MPs reference spectra and the associated 7 spectral filters (bright areas correspond to selected wavenumbers) (b) White light image and ground truth indication of the different MPs present in the field of view (FoV). The dashed square indicates the field of view of the Raman microscope as seen in Fig. 4(d). (c) Projection maps obtained from filter measurements. (d) CRT final classification imaging (PS: red; PET: yellow; PU: cyan, PE: purple). Acquisition speed: 2 ms/pix by applied filter, FOV: 300 *μ*m × 300 *μ*m, scale bar: 50 *μ*m.

Following the recording of the reference spectra (Fig. 4(a)), the construction of binary filters was carried out for all the spectra of six plastic polymers coming from the marine environment (Fig. 4(a)). These different filters were projected onto a sample that is a mixture of MPs polymers (PS, PP, PET, PU, PA, and PE – see Fig. 2) deposited on a CaF_2_ coverslip. The chosen imaging area corresponds to a FOV of 300 ×300 *μ*m (Fig. 4(b)). Note that for ground truth determination, check was performed on the spectra of each MP bits present in the imaging region.

After the projection of each filter on the chosen imaging region, the projection maps are obtained, *i.e.* the photons number received per pixel for each filter (Fig. 4(c)). Fig. 4(d) shows the result of the classification where an RGB filter has been applied to highlight the different plastic polymer type. A very good agreement is found with the ground truth (Fig. 4(b)) with PET in yellow, PS in red, PE in purple, PU in light blue, PP in dark blue and CaF2 in black. Note that although the PA filter was applied, none PA bits are found in this region of interest.

### 3.3 High-speed CRT imaging of MP coming from the marine environment

**Fig. 5:**
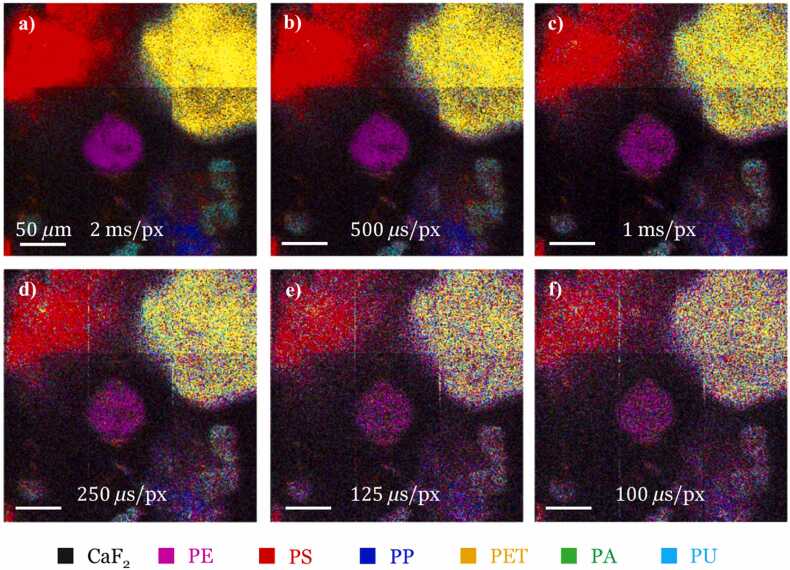
CRT imaging performed at different pixel dwell time. (a) 100 *µ*s/pix/filter, (b) 125 *µ*s/pix/filter, (c) 250 *µ*s/pix/filter, (d) 500 *µ*s/pix/filter, (e) 1 ms/pix/filter, (f) 2 ms/pix/filter. FOV: 300 *µ*m × 300 *µ*m (300 pixels × 300 pixels); scale bar: 50 *μ*m.

To investigate the ability of CRT setup to perform fast imaging of MPs, Fig. 5 compares the CRT image quality obtained with pixel dwell time ranging from 100 *µ*s/pix/filter to 200 *µ*s/pix/filter. The considered MPs sample and the region of interest is the same as in Fig. 4. At 250 *µ*s/pix/filter it is still possible to distinguish the five MPs polymers PE, PS, PP, PET and PU. This probably sets the speed limit on this type of sample to access a decent signal-to-noise ratio for MPs detection. A quick statistic test applied to data (see supp info paragraph 4) showed a loss of information of more than 50% when scanning speeds exceeded 250 *µ*s/pix/filter. This speed of 250 *µ*s/pix applied per filter represents a total time of 2.6 min to build an image with a FOV of 300 *µ*m × 300 *µ*m (with pixel dwell time of 1.75 ms).

For increasing pixel dwell time, the error probability decreases and the classifier reveals the accurate chemical species at each pixel. As a consequence, with increasing pixel dwell time, more pixels take the same color on macroscopic areas corresponding to a MP type. In the supplementary information Figure S2, validation of the CRT identifications of MPs for five polymers is conducted by recording full Raman spectra (as CRT can be switched to conventional Raman by sequentially scanning each pixel columns on the DMD). This corroborates the different chemical species identified by CRT.

### 3.4 CRT detection of MP from the marine environment

**Fig. 6:**
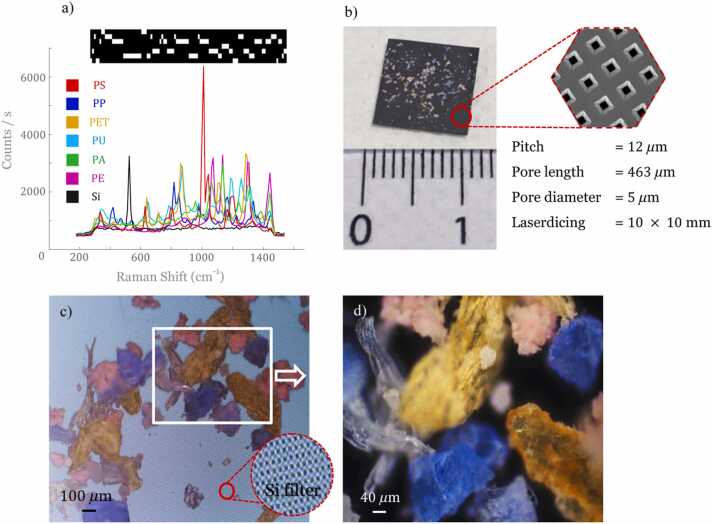
(a) Reference spectra of the different MPs polymers with that of silicon filter (Si, in black) from which the binary orthogonal spectral filters have been constructed. (b) Image of the silicon filter (size: 10 mm x 10 mm) after passing it through an aqueous medium containing a mixture of 6 MPs. (c) View from the microscope camera a FOV of the different MP polymers collected by a silicon substrate. A magnification is performed on the region of interest (white frame in c) to obtain (d).

To evaluate the ability of CRT to be used in the field to detect and quantify MPs, a silicon filter has been used (131675 W14 - SmartMembranes) (MakroPor_002.pdf, 2022) as the substrate on which the MPs are deposited. These types of filters are commonly used to filter particles in liquid. This silicon filter has pores with a diameter of 5 *μ*m, a thickness of 463 *μ*m and a pore spacing of 12 *μ*m. These type of filters guarantee sufficient transparency for the wide mid-IR of 4000–600 cm-1 (Käppler et al., 2015) and their mechanical properties make them good candidate for field application. In order to use this silicon support in MPs analysis, its measured Raman spectrum has been added to the pool of pure MPs Raman spectra (Fig. 4(a)) and ran the algorithm to design the 7 binary orthogonal filters that will enable the CRT measurements (Fig. 6(a)). To build the sample, the MPs have been directly incorporated into pure water. Then, the silicon filter was directly immersed in the aqueous medium containing the six different MPs polymers (PE, PET, PA, PS, PU, and PP). The silicon filter was then dried in open air and the MPs found themselves naturally attached to its wall (Fig. 6(b)). Two images were taken with two different magnifications to obtain a view of the sample from which to apply MPs classification. A magnification from Fig. 6(c), in the white boxed area, was used to obtain the image shown in Fig. 6(d).

### 3.5 MPs CRT imaging over a 1 mm² silicon filter area

**Fig. 7:**
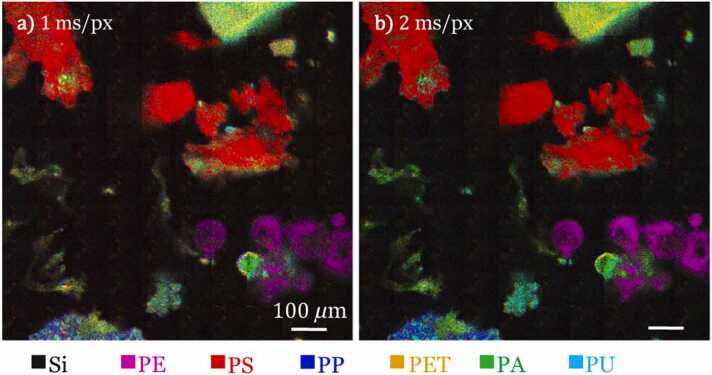
CRT imaging of MPs performed on a silicon filter substrate, (a) 1 ms pixel dwell time and (b) 2 ms pixel dwell time, for each of the seven spectral filters applied. Scale bar: 100 *μ*m, total FOV = 1 mm × 1 mm (1000 pixels × 1000 pixels).

CRT imaging was performed over a large (1 mm^2^) area of the Si substrate containing the collected MPs. Performing MPs detection and quantification over large areas is necessary to perform relevant statistical analysis. Fig. 7 compares the 1 mm × 1 mm FOV CRT images quality obtained with a pixel dwell time of (a) 1 and (b) 2 ms/pix/filter (7–14 ms total pixel dwell time, respectively) for each of the seven filters applied. This scan speeds have been selected to have superior image qualities as compared to those obtained with faster scanning speeds (see Fig. 5). These 1 mm^2^ images (1000 pixels x 1000 pixels) are obtained after total acquisition times of 2 h 10 min and 4 h 32 min, respectively. This is to compare to the 500 ms pixel dwell time reported in the literature that lead to 10 s to 100 s of hours acquisition time (depending on the pixel number) (Araujo et al., 2018, Sobhani et al., 2019, Käppler et al., 2015). The various types and shapes of the MPs present in the field of view can be clearly seen using CRT imaging.

## 4 Discussion

The ability of compressive Raman technology (CRT) to image and quantify micro-plastics (MPs) originating from natural marine environments has been successfully evaluated. CRT is different than conventional Raman imaging (that record the full Raman spectra) because CRT uses a priori known spectral information to design a limited (one per MP chemical species) set of spectral filters that can be used to perform fast imaging using a single pixel detector. Despite the countless difference that can exist between each type of plastic due to their added additive, there are only about ten major microplastic polymers released in the marine environment. This suits perfectly the CRT technology. Indeed, too many species would require many spectral filters that would make CRT non-optimal as compared to a conventional Raman spectrometer. Because there is a limited (< 10) number of relevant MPs, there is a limited number of projection images (each of them corresponding to a specific spectral filter) that need to be acquired to reveal all the relevant information to image, detect and classify MPs. CRT can image and classify MPs with a pixel dwell time as short as 250 *µ*s/pix/filter where each species could be detected and correctly identified. With 7 polymer species of MPs, this brings down the overall illumination pixel dwell time to 1.75 ms, a time which is ×10–100 shorter that what is commonly used in MPs identification using conventional Raman spectroscopy and imaging (Araujo et al., 2018, Käppler et al., 2016). This fast imaging and classification speed can be advantageously used to image large surface areas and images were shown over 300 μm × 300 μm field of view (acquired in 21 min, and down to 2.6 min for pixel dwell times of 2 ms/pix/filter and 250 us/pix/filter, respectively) and over 1 mm × 1 mm acquired in 2 h on a silicon filter that is used to filter MPs in liquids. This time is to be compared with the 12.5 h reported into the literature to image similar areas of MPs with the same resolution (1 μm) and a pixel dwell time of 0.5 s (Araujo et al., 2018, Sobhani et al., 2019). Beside this key speed advantage, CRT has been successfully used to image MPs coming from the natural environment where most samples are pigmented. Quite interestingly the reference MPs spectra and their associated computed binary filters were taken from uncolored MPs (i.e. synthetic uncolored MPs with no additives). The reference spectra on the uncolored MPs are shown in Fig. S2 c whereas the spectra from the marine environment samples are shown in Fig. S2 b. There are clearly some fluorescence backgrounds on some of the marine MPs (PET, PU, PP) but quite remarkably the binary filters taken from the uncolored, unpigmented, background free reference MPs are working to classify the marine MPs from natural environment. This shows the relative robustness of the binary filters that are built with reference background free MPs to address larger and wider types of similar MPs in terms of additives, colors and fluorescence background. This has of course some limits and we fail in imaging black Polyvinyl chloride (PVC) particles that always exhibit large and unstable fluorescence background followed by irreversible photo-damages.

However, the samples used in this paper are less complex than real samples of sediment or organic matter. In fact, even after pre-treatment, these samples may still contain clay-like particles, shell fragments, organic debris, etc. In supp info Fig. S5 we have conducted a simple experiment to address this complexity. We demonstrate that despite the presence in the sample of an unknown (organic) species (in this case a leaf, whose spectral information was not taken into account in the initial model to build the binary filters), only the species initially considered to build the binary filters are present in the final Raman classification image. This demonstrate the relative resilience of CRT to be contaminated by unknown Raman species that are not taken into account in the design of the binary filters.

Some of the MPs detected in Fig. 5, Fig. 7 show different level of classification quality. This is probably due to the limited ≈10 *µ*m z resolution of CRT microscope and the 3D nature of the MPs samples that provide an optimal SNR only for MPs located in the same plane. 3D imaging is possible using a z scanner at the price of a longer image acquisition time. Throughout this work, orthogonal binary spectral filters were used, these filters use sets of wavelengths that do not overlap, *i.e.* a wavelength used in one filter is not used in another filter in the set. The orthogonality requirement is not mandatory in the CRT context, as demonstrated in previous studies (Scotté et al., 2018, Grand et al., 2022). However, it was observed that the orthogonality constraint proved beneficial in the context of MPs imaging and quantification, likely owing to the fluorescence background.

## 5 Conclusion

We have demonstrated that compressive Raman can rapidly image and classify microplastics (MPs) with a shortest pixel dwell time of 250 µs per projection filter. This reduces the pixel dwell time to 1.75 ms to detect the six major MPs, which is >50 times faster than previously reported work using conventional Raman. When compared to other techniques, CRT has several advantages. First, like most optical techniques, it employs relatively light and inexpensive technology compared to mass spectrometry. This is beneficial when implemented in conventional laboratories or deployed for underwater investigations (Liu et al., 2021). Second, CRT is well-suited for MPs detection because (i) the Raman spectra of MPs are known, and (ii) there are fewer than 10 major MPs to detect, requiring the sequential display of fewer than 10 binary filters to complete the detection of the MPs. Third, because CRT uses a single-pixel detector that is shot-noise-limited and has a fast (MHz) response time, it allows for fast imaging and provides MPs morphological information, with pixel dwell time limited only by the Raman signal. These advantages make CRT superior to mass spectrometry, FTIR, and conventional Raman for rapid detection and imaging of MPs. Further improvements to increase imaging speed could be envisioned, such as 'region of interest predeterminations' using white light images, sub-sampling (decreasing spatial resolution), or flow detection, which would reduce the image to a line or even a point. These improvements could further establish CRT as a game-changer for MPs imaging and quantification in environmental applications.

## CRediT authorship contribution statement

**Hervé RIGNEAULT:** Writing – review & editing, Writing – original draft, Supervision, Methodology, Funding acquisition, Conceptualization. **Enora Prado:** Writing – review & editing, Resources, Methodology. **Maria El Rakwe:** Resources. **Olivier Fauvarque:** Writing – review & editing, Resources, Investigation. **Camille Scotté:** Software, Formal analysis. **Clément Grand:** Writing – original draft, Visualization, Validation, Investigation.

## Declaration of competing interest

The authors declare no conflict of interest

## Data Availability

Data will be made available on request.
