# Supplementary material for: Fast compressive Raman micro-spectroscopy to image and classify microplastics from natural marine environment
Source: Environ Technol Innov. 2024 May;34:103622. doi: 10.1016/j.eti.2024.103622 (PMC11066848; doi:10.1016/j.eti.2024.103622)
Supplement: Supplementary file 1 — Supplementary material [file mmc1.docx]

Supplementary material

Fast compressive Raman micro-spectroscopy to image and classify microplastics from natural marine environment.

Clément Grand^1^, Camille Scotté^1,2^, Énora Prado^3^, Maria El Rakwe^3^, Olivier Fauvarque^3^, and Hervé Rigneault^1*^.

^1^Aix Marseille Univ, CNRS, Centrale Marseille, Institut Fresnel, Marseille, France

^2^INRAE, UMR ITAP, 361 Rue Jean François Breton, 34090 Montpellier, France

^3^Ifremer, RDT Research and Technological Development, F-29280 Plouzané, France

**1- Detailed CRT experimental setup**


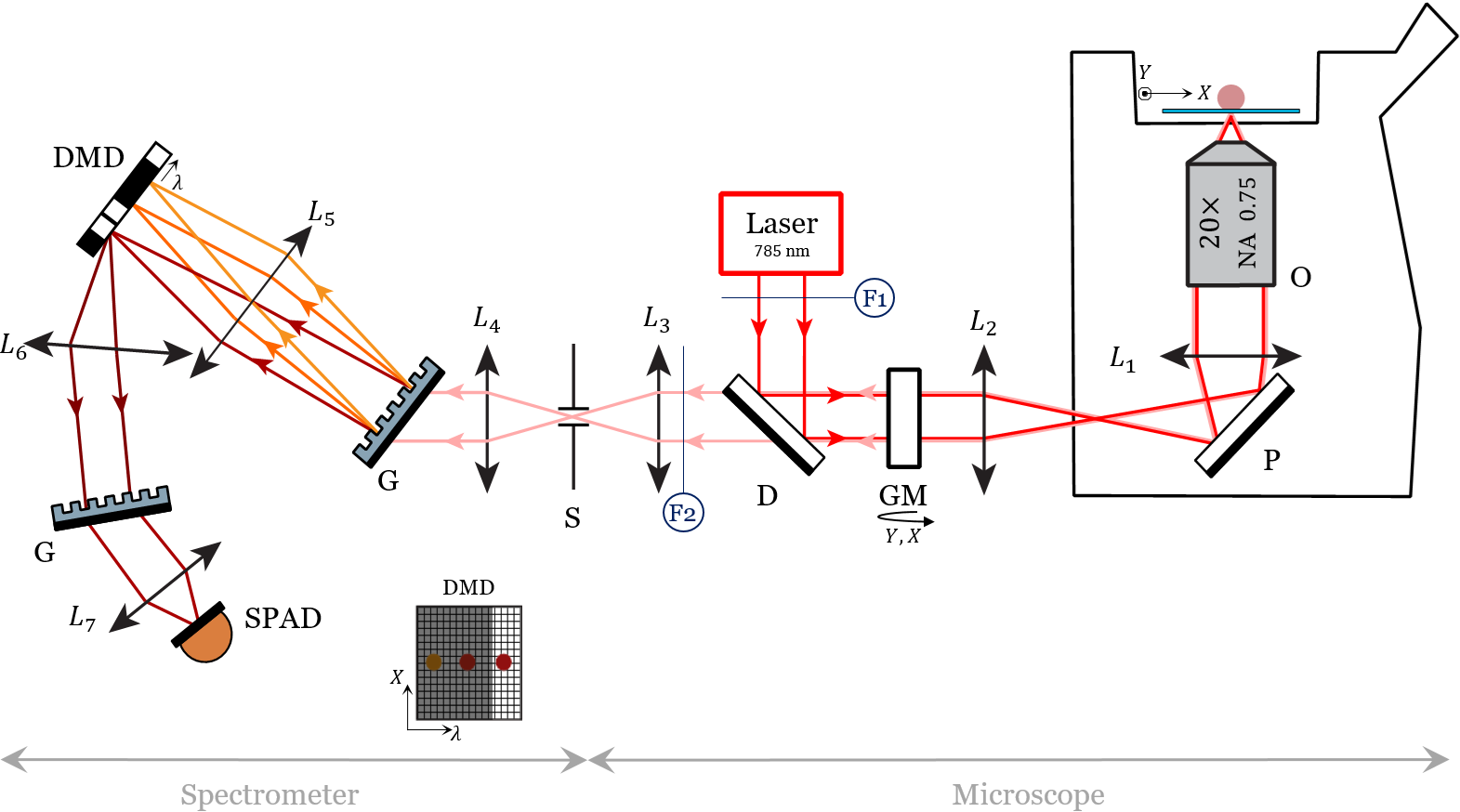


**Figure S1:** Compressive Raman technology CRT experimental setup built around a commercial microscope stand (Nikon Eclipse TiU) adapted from [37]. P: prism, GM: galvanometric-mirrors, D: Dichroic mirror, S: slit, G: grating, SPAD: single-photon avalanche photodiode, F1: laser line filter (LL01-785-12.5, Semrock), F2: notch filter (HSPF785.0 – Kaiser). L1: tube lens (200 mm), L2: scanning lens (50 mm), L3 - L7: achromatic doublets with focal lengths 100, 125, 100, 100, 75 mm, respectively.

GM: Cambridge Instrument, D: LPD02-785RU - Semrock, G: T-1200-850s - Lightsmyth, SPAD: IDQ ID120-500-800-ULN, DMD: Vialux V-650L NIR, Objective: Nikon CFI Apo Lambda. The laser is a 785 nm CW from IPS (IPS-L2K0785SD0090B-ISTH-L) providing 100mW of output power.

Note that after the DMD the setup uses a second grating to recombined the colors in a single angular direction to enable the focus on the 500 *μ*m SPAD active detector surface.

**2- Raman spectra confirmation of the pure species retrieved by CRT**

LA

LA

LA

2 ms/px/species

FOV : 300 *µ*m²

Average of 10 spectra from the same color pixels

Counts

Raman shift (cm^-1^)

0

400

800

1200

1600

a. Hyperspectral data cube

c. Reference spectra


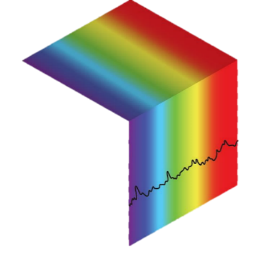


$x$ (*µ*m)

$y$(*µ*m)

300

300

$\lambda$ (nm)


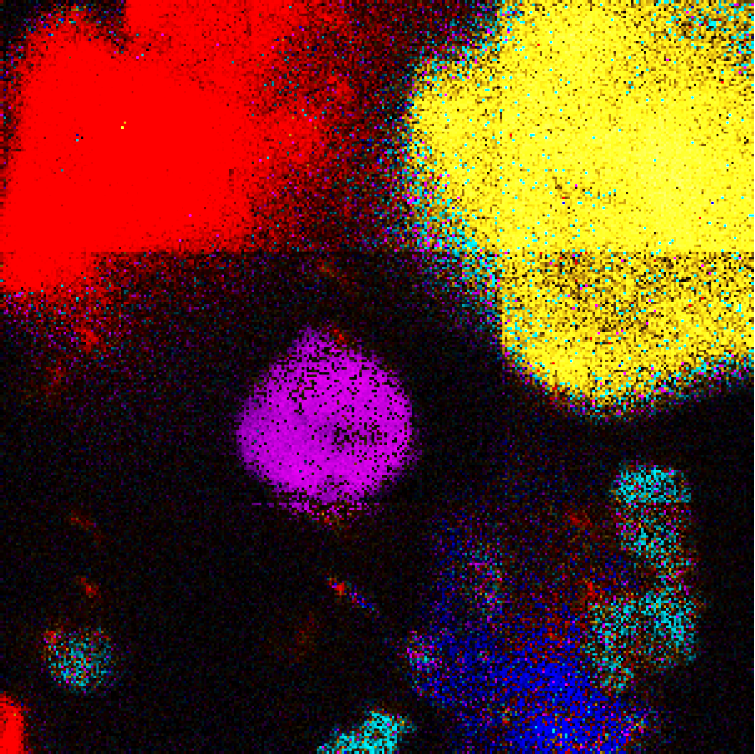


**400**

1000

1500

**500**

2500

1000

2000

**500**

1500

2500

**0**

2000

4000

6000

**0**

2000

**120**

200

280

380

**150**

450

300

**100**

200

600

**100**

300

600

900

0

400

800

1200

PS

PET

PU

800

**220**

260

PP

PE

400

1600

b. Averaged Raman spectra

Counts/s

d. MPs spectra (ground truth)

Intensity (a.u)

Raman Shift (cm^-1^)

200

600

1000

1400

**0**

0.5

1

1

**0**

0.5

1

**0**

0.5

1

**0**

0.5

1

**0**

0.5

**Figure S2:** **(a)** Once the pure species had been recovered by CRT (Fig. 4 and 5) (scan speed: 2 ms/pixel/filter), we studied the complete Raman spectra of each species by scanning the DMD pixel column one by one. To do this, we acquired a hyperspectral image with the DMD. Indeed, CRT can be switched to conventional Raman by sequentially scanning each pixel columns on the DMD. For a better SNR we averaged the full Raman spectra over 10 pixels belonging to the same pure specie on the CRT image. **(b)** The complete averaged Raman spectra obtained from the hyperspectral cube (b.PS (in red), b.PET (in yellow), b.PE (in pink), b.PU (in turquoise), b.PP (in blue)) are compared **(c)** with the pure sample reference spectra (from Fig. 4 (a)) obtained with an integration time of 100 ms/pixel/species, **(d)** with the spectra measured by Raman spectrometry (HR800 spectrometer equipped with 785 nm excitation laser and 300 lines/mm grating after plastics fragmentation - specific background was subtracted from each spectrum) . Clearly CRT is able to retrieve the correct chemical species for each pixel.

**3- CRT theory, filter design and classification**

We provide a brief overview of the mathematical method developed in [26] [35] [38] and focus on quantification (species proportions estimation).

***Spectral mixing model:***

We assume a sample with spectrum $y$ (measured over a certain frequency bandwidth ν of $K$ spectral bases) consists of a mixture of $Q$ chemical species of known spectra $S_{i}$. Under a linear mixing assumption, we can write:

|  | $y\left( \nu\right)= \sum_{i=1}^{Q} z_{i}S_{i}\left( \nu\right)$ | (S.1) |
| --- | --- | --- |

Where $z_{i}$ are the unknown proportion coefficients.

Let $M$ binary filters be sequentially displayed onto the DMD with $M\geq Q$. If the measurements are limited by Poisson noise, the measured number of photons $n_{m}$ through filter $F_{m}$ is modeled as a Poisson process ($m=1\ldots M$). For filter$F_{m}$, the mean number of measured photons $\mu_{m}=\left\langle n_{m} \right\rangle$ is a projection of the filter $F_{m}$ onto the sample spectrum y for a certain exposure time $\tau_{m}$:

|  | $\mu_{m}= \tau_{m}\sum_{k=1}^{K} F_{m}\left( \nu_{k} \right)y(\nu_{k})$ | (S.2) |
| --- | --- | --- |

Which, with equation (S.1), leads to

|  | $\mu_{m}=\sum_{i=1}^{Q} G_{mi}z_{i}$ | (S.3) |
| --- | --- | --- |

(Or, in the matrix form: $\boldsymbol{\mu=Gz}$).

Where

|  | $G_{mi}=\tau_{m}\sum_{k=1}^{K} F_{m}\left( \nu_{k} \right)S_{i}(\nu_{k})$ | (S.4) |
| --- | --- | --- |

The two above equations state that the mean number of measured photons $\mu_{m}$is the projection of the filters $F_{m}$ during time $\tau_{m}$onto the pure spectra $S_{i}$ weighted with their relative proportion coefficients $z_{i}$.

***Binary Filter design***

To design the filters, we consider an unbiased estimator whose precision is given by the covariance matrix $\boldsymbol{\Gamma}= \left\langle\delta\hat{z} \delta\hat{z}^{T} \right\rangle$, where $\hat{z}$ is an estimation of $z,$ $\delta\hat{z}=\hat{z}-\left\langle\hat{z} \right\rangle$ and **^T^** is the transpose operation.

The $M$ filters can then be determined in order to optimize a figure of merit deduced from $\boldsymbol{\Gamma}$. The figure of merit used in the algorithm in (1) is the trace of the Cramer-Rao lower bound (CRB) matrix. This CRB is a lower bound on the variance of any unbiased estimator and is given by [33]:

| ${\boldsymbol{[CR}\boldsymbol{B}^{\boldsymbol{-1}}\boldsymbol{]}}_{ij}=\left[ \boldsymbol{I}_{\boldsymbol{F}} \right]_{ij}= \sum_{m=1}^{M} \frac{G_{mi}G_{mj}}{\mu_{m}}$ | (S.5) |
| --- | --- |

Where $\boldsymbol{I}_{\boldsymbol{F}}$ is the information matrix. The above equation shows that the precision is a function of the binary filters, the proportions and the exposure time.

The implemented algorithm for filters design consists in a simple numerical optimization technique: a set of values $(m,k)$ is randomly chosen and the value $F_{m}(k)$ replaced by $1-F_{m}(k)$ if $trace(\boldsymbol{CRB})$ is decreased.

Following the recording of the reference spectra, the construction of orthogonal binary filters was carried out optimally for all the spectra of the six plastic polymers (Fig. 4(a)). Indeed, the use of non-orthogonal binary filters in combination with each other has shown much less convincing results than orthogonal binary filters. We will not go into more detail here but a quick comparison of some results is shown in Appendix of [36].

***Classification algorithm***

***
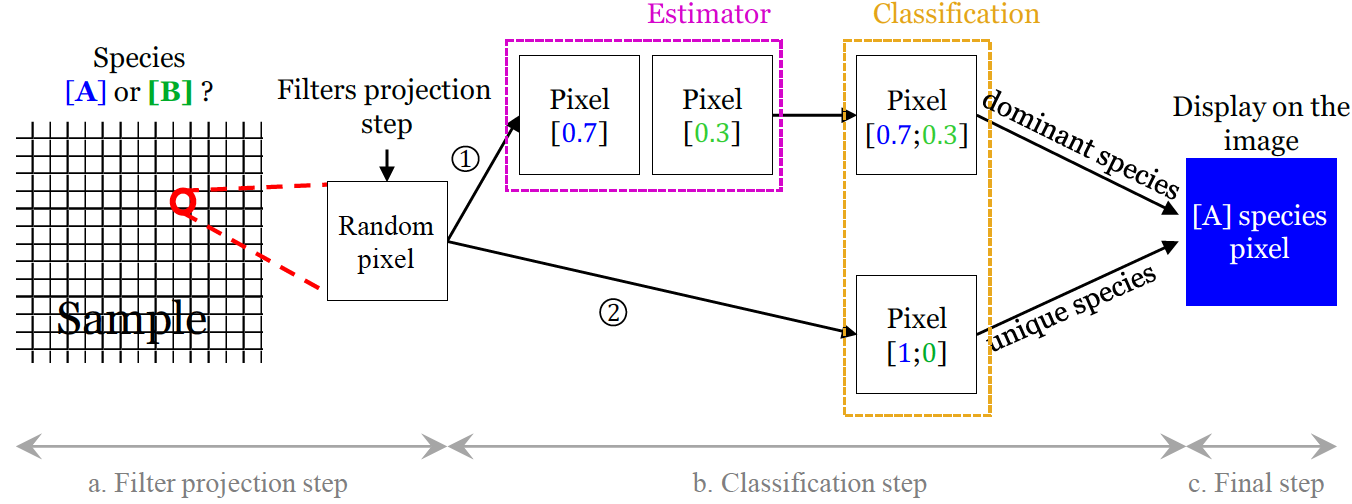
***

**Figure S3:** Simplified illustration of the two classification methods used. **(a)** Represent a random pixel on which several filters will be projected to know which color (species) it will represent. **(b)** Is the classification step applied **(1)** with an estimator or **(2)** directly. **(c)** Is the display step of the color selected by the classification.

From Fig. S4, we saw that a direct application of the classifier allows us to skip the estimation step we used in [26] [30] [36] (Fig. S4(b)). This classifier is based on the different Raman signal returns (number of photons:$x_{1}$, $x_{2}$) obtained after the application of the different spectral filters depending on the species A and B. The probability of species A or B for each pixel in the sample is then compared. The classifier then chooses the species to be displayed in each pixel of the sample according to the highest probability of identification (equation below). Therefore, for a single pixel, we now have one and only one data corresponding to the species whose associated color is displayed.

Choose the highest probability for each pixel

$$[B]$$

$$[A]$$

Green

Pixel

Blue

Pixel

$$\mathcal{P} \left( \left[ x_{1} x_{2} \right] | [A] \right)$$

$$\mathcal{P} \left( \left[ x_{1} x_{2} \right] | [B] \right)$$

**4- Statistical limitations of CRT algorithm for sampling specific MPs**

300 *µ*m $\times$ 300 *µ*m

50 *µ*m

2 ms/px

a. Raman classification map

b. Scan speed maps for yellow pixel class

c. Yellow pixel class evolution from

the largest yellow cluster

**Figure S4:** **(a)** The classification map of our plastic samples with an acquisition speed of 2 ms/px, **(b)** the same FOV where only the yellow pixels are represented as a function of the different scan speeds, and **(c)** a curve representing the evolution of the number of yellow pixels contained in the largest yellow zone represented in the image as a function of the different scan speeds. The different acquisition speeds range from 2 ms/px to 100 *µ*s/pix. All the images shown have a FOV of 300 *µ*m $\times$ 300 *µ*m and the scale bar is equal to 50 *µ*m.

In this context, we aim to determine the acquisition speed limit that should not be exceeded when analyzing MPs (Microplastics) samples with our CRT (Compressive Raman Technology). When we analyze and isolate a specific pixel color in our classification image (in this case, yellow, corresponding to PET as shown in Figure S5(b)), we observe that the number of yellow pixels varies with the acquisition speed.

To investigate this potential loss of information, we considered our image captured at the highest scan speed (2 ms/px) to represent 100% of the revealed information. Figure S5(c) illustrates the state of various yellow pixels within the largest yellow cluster (located at the top-right of the image). Depending on the acceleration of our scan speed, we notice a change in the category of these pixels, indicating a shift in color and, consequently, the transmission of different information to the observer.

The curve in the graph delineates the progression of these yellow pixels in this area and enables us to interpret the potential loss of information resulting from the increased scan speed compared to the data obtained at 2 ms/px. At scan speeds exceeding 250 *µ*s/px, the information differs by more than 50% (reaching 72% at 125 *µ*s/px) from the original data obtained at 2 ms/px. This suggests a significant loss of information, urging us not to exceed a scan speed of 250 *µ*s/px, which already signifies a 50% loss of information.

**5- Demonstration of a simple CRT model with an unknown specie**


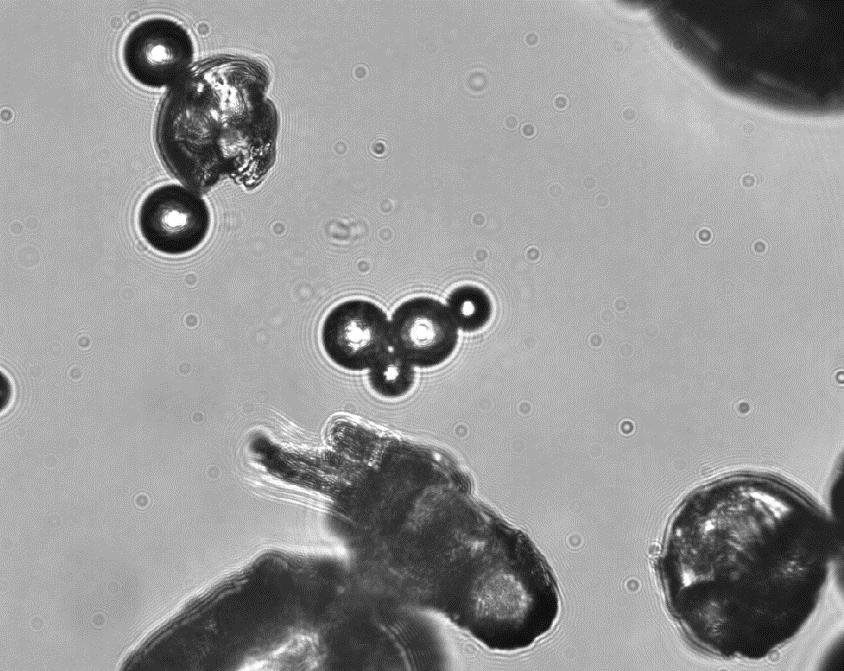


a. Sample

b. Filtered measurements

FOV

Leaf

PMMA

PS

c. Classified image

30 *µ*m

d. Hyperspectral data

20

60

100

20

60

100

2

1

0

$\times$10^4^

Filter 1

30 *µ*m

6

4

2

$\times$10^4^

Filter 2

10

6

2

$\times$10^4^

Filter 3

30 *µ*m

**Figure S5:** **(a)** Sample seen from a microscope camera. **(b)** Projection map of the three different spectral filters from the image seen in (a) where the exposure time is 500 *µ*s/pixel/filter. **(c)** Classification map after applying the algorithm, described in [38], on the filtered measurements shown in (b). **(d)** Hyperspectral data from a pixel representing the spectrum measured on a verbena leaf (the graph shows the intensities (y) and cm^-1^ (x)). For each image, the scale bar is 30 *µ*m and the FOV is 100 *µ*m $\times$ 100 *µ*m (100 pixels $\times$ 100 pixels).

We conduct here a simple experiment to demonstrate that despite the presence of an unknown (organic) species (in this case a leaf, whose spectral information was not taken into account in the initial model to build the binary filters), only the species initially considered to build the binary filters are present in the final Raman classification image The model used here is exactly the same as that shown in Fig. 3, i.e. the filters were constructed from the spectra of the two types of plastic beads, PS (red) and PMMA (green), and the background (the CaF2 coverslip). The beads were placed in a sample with microparticles of dried verbena leaf (Fig S5 (a)). This verbena fragments are used to simulate an unexpected species in the measurements. The filtered measurements observed in Fig S5 (b) represent the photons collected after the display of the three filters (constructed from the Raman spectra of PMMA, PS and CaF2). The part of the verbena leaf, visible at the bottom left of the FOV, stands out in all three filters, but particularly in filter 2. However, this unexpected species is not displayed in the final Raman detection and identification image shown in Figure S5 (c). Only the plastic beads, whose spectral information were taken into account at the start of the measurements, show up correctly and in the right color.

Although some of the colored pixels are identifiable from the black background at the location of the verbena leaf, they have been well filtered from the classification process. Figure (d) shows the spectral information recorded on these isolated color pixels. It turns out that this does not correspond to the spectral identity of a verbena leaf. This spectral signal probably corresponds to a fluorescence background of the leaf.
